# Intra- and Early Post-Operative Factors Affecting Spinal Cord Ischemia in Patients Undergoing Fenestrated and Branched Endovascular Aortic Repair

**DOI:** 10.3390/jcm13133978

**Published:** 2024-07-08

**Authors:** Allegra Doering, Petroula Nana, José I. Torrealba, Giuseppe Panuccio, Constantin Trepte, Viorel Chindris, Tilo Kölbel

**Affiliations:** German Aortic Center, Department of Vascular Medicine, University Heart and Vascular Center UKE Hamburg, 20251 Hamburg, Germany; a.doering@uke.de (A.D.); jitorrealba@gmail.com (J.I.T.); g.panuccio@uke.de (G.P.); c.trepte@uke.de (C.T.); v.chindris@uke.de (V.C.); tilokoelbel@googlemail.com (T.K.)

**Keywords:** fenestrated endovascular aortic repair, branch endovascular aortic repair, spinal cord ischemia, glucose, hemoglobin, paraplegia

## Abstract

**Background**: Spinal cord ischemia (SCI) is a severe complication after fenestrated/branched endovascular repair (f/bEVAR). The underlying causes of SCI are still under investigation. This study aimed to evaluate intra- and early post-operative parameters that may affect SCI evolution. **Methods:** A single-center retrospective analysis was conducted including SCI patients with complete anesthesiologic records (1 January 2011 to 31 December 2023). Values of intra-operative glucose, hemoglobin, lactate, activated clotting time (ACT), and the need for transfusion were collected. The cohort was compared to a matched cohort of non-SCI patients. **Results:** Fifty-one patients with SCI and complete anesthesiologic records were included (mean age: 69.8 ± 6.2 years; 39.2% male). Intra-operative glucose value < 110 mg/dL (AUC: 0.73; sensitivity 91%, specificity of 83%) and hemoglobin value > 8.5 mg/dL (AUC: 0.61; sensitivity 83%, specificity 78%) were protective for Grade 3 SCI. Twenty-three patients with SCI were matched to 23 patients without SCI. SCI patients presented significantly higher glucose levels intra-operatively (glucose mean value: SCI 150 ± 46 mg/dL vs. non-SCI: 122 ± 30 mg/dL, *p* = 0.005). ACT (SCI 259 ± 31 svs. non-SCI 288 ± 28 s, *p* = 0.001), volume input (SCI 4030 ± 1430 mL vs. non-SCI 3020 ± 113 mL, *p* = 0.009), and need for transfusion (SCI: 52.5% vs. 4.3%, *p* < 0.001) were related to SCI. Higher glucose levels were detected among patients with SCI, at 24 (SCI: 142 ± 30 mg/dL vs. non-SCI: 118 ± 26 mg/dL, p=0.004) and 48 h (SCI: 140 ± 29 mg/dL vs. non-SCI: 112 ± 20 mg/dL, *p* < 0.001) post-operatively. **Conclusions:** SCI is a multifactorial complication after f/bEVAR. Intra-operative and early post-operative glucose levels may be related to SCI evolution. Targeted glucose < 110 mg/dL may be protective for Grade 3 SCI.

## 1. Introduction

Spinal cord ischemia (SCI) is one of the most devastating complications affecting patients undergoing fenestrated/branched endovascular aortic repair (f/bEVAR) [1,2,3,4,5]. SCI affects highly not only the health system but mainly patient survival and quality of life, with 75% of them surviving only a year after symptom evolution [5]. Especially for patients presenting with paraplegia (Grade 3 SCI), the estimated survival is limited by almost 3 years compared to patients with non-SCI or SCI Grades 1 and 2, highlighting the need for SCI Grade 3 prevention, early recognition, and management [6]. 

While the rates of SCI have decreased over time, patients with extensive thoracoabdominal aortic aneurysms (TAAAs) still represent a group at high risk for SCI development, with rates up to 20% [2,6,7]. The mechanisms leading to SCI are not yet fully understood, with extensive aortic disease and its subsequent coverage, occlusion of collateral networks, and restricted flow in the left subclavian artery (LSA) and hypogastric arteries being related to worse outcomes [8,9].

Protective measures, including collateral network preservation, preconditioning with embolization of segmental arteries, and staged procedures, seem to decrease the risk for post-operative SCI [10,11]. The role of cerebrospinal fluid drainage (CSFD) is still to be clarified, with conflicting findings in the literature [12]. Intra-operative factors, including hemoglobin level, blood loss, blood pressure, need for transfusion, and glucose level have been less investigated in clinical terms while they seem of increased importance for SCI evolution [3,8,13,14,15].

This study aimed to evaluate the role of intra- and early post-operative parameters among SCI patients, focusing on Grade 3 SCI, and compared the findings with propensity-matched patients without SCI. 

## 2. Materials and Methods

### 2.1. Study Design

A single-center retrospective analysis was conducted according to the STrengthening the Reporting of OBservational studies in Epidemiology (STROBE) statement [16]. All patients with SCI and complete intra-operative anesthesiologic records, managed for thoracoabdominal, para-, and juxtarenal aneurysms (degenerative or post-dissection), using f/bEVAR (custom-made or off-the-shelf devices), from 1 January 2011 to 31 December 2023, were included. This cohort was matched to a cohort of patients managed with f/bEVAR but without post-operative SCI. This study complied with the Declaration of Helsinki and no approval was required from the local ethics committee according to the current state law due to its retrospective design and unidentifiable information. 

### 2.2. Inclusion Criteria

Only patients managed with the Zenith platform (Cook Medical, Bloomington, IN, USA) with custom-made fenestrated or branched devices as well as off-the-shelf devices were considered eligible. Devices with fenestrations, outer and/or inner branches, or a combination of them were included. The extent of the disease was not a criterion for exclusion and patients with juxta-, pararenal, and thoracoabdominal aneurysms were included. Elective and urgent (symptomatic and ruptured aneurysm) cases were included. Patients who presented with SCI after f/bEVAR represented the main cohort of the current analysis, regardless of the grade of SCI. However, the analysis was performed only in patients with available detailed anesthesiologic reports, which permitted investigation of the factors of interest. Patients without complete records, hampering the extraction of intra- and early post-operative parameters, were excluded. Patients managed with standard endovascular abdominal (EVAR), surgeon-modified grafts, other platforms of fenestrated or branched devices, or a parallel graft technique were excluded. Patients managed with fenestrated or branched aortic arch devices were not included if no distal extension into the thoracoabdominal aorta was present. 

### 2.3. Data Collection

Pre-, intra-, and post-operative details of patients were collected [17,18]. Intra-operative information included the type of repair (f/bEVAR), extent of coverage according to the Society of Vascular Surgery (SVS)’s reporting standards’ zones, preservation of collaterals (LSA and hypogastric arteries), need for additional procedure for collateral preservation, intercostal or lumbar artery embolization (previous or concomitant), and use of CSFD (prophylactic or therapeutic) [19]. Technical success was also noted. 

Intra-operative blood values for glucose (maximum, minimum, and mean value); hemoglobin (maximum, minimum, and mean value); need for transfusion, including transfused volume (mL) and input volume (mL); lactate (maximum, minimum, and mean value); administrated heparin in international units (IU); and activated clotting time (ACT; maximum, minimum, and mean value) were collected. Data on mean systolic blood pressure, in addition to maximum and minimum values, were noted. Blood pressure was calculated invasively using an arterial line.

For the first 48 post-operative hours, the values of glucose (mean value for the 1st and 2nd post-operative day), hemoglobin (mean value for the 1st and 2nd post-operative day), creatinine (mean value for the 1st and 2nd post-operative day), and the need for transfusion were recorded. Post-operative adverse events including 30-day mortality, myocardial infarction (MI), stroke, acute kidney injury (AKI), and bleeding, with or without the need for re-intervention, were also collected. The length of stay in the intensive care unit (ICU) and the total length of hospital stay (LOS) were recorded. 

### 2.4. Definitions 

The SVS reporting standards were used for definitions [17]. Any post-operative new-onset neurologic deficit of the lower limbs, including any paraplegia or paraparesis, that could not be related to other pathologic mechanism, was characterized as SCI, while grading was performed according to the reporting standards (Grade 0: no deficit; Grade 1: minimal sensory deficit; Grade 2: paraparesis; and Grade 3: paraplegia) [15]. The evolution of SCI, complete recovery, partial recovery, and non-recovery, as well as the time of onset (early: within 24 h; late: after 24 h) were also investigated according to reporting standards [17].

### 2.5. Outcomes 

Peri-operative medical factors that affected SCI evolution after f/bEVAR, with emphasis on Grade 3, were the primary outcome. A comparative matched analysis was performed to investigate the role of medical factors in SCI evolution. 

### 2.6. Statistical Analysis 

Continuous data were reported using the mean ± standard deviation and non-continuous data were expressed as median values with the associated interquartile range (IQR). Categorical data were expressed as absolute numbers or percentages. Independent two-sample *t*-test was used for normally distributed continuous variables, and the Wilcoxon rank sum test was used for non-normally distributed continuous and ordinal variables. Chi-square or Fisher’s test was used for comparisons of categorical variables. No correction for multiple hypothesis testing was applied. A ROC curve was constructed for risk assessment to define a cut-off value for Grade 3 SCI evolution. A propensity score model was created incorporating patient age and sex; known (for at least for 3 months) chronic renal disease of ≥stage 3 according to Kidney Disease Improving Global Outcomes (KDIGO) criteria; ASA score (1 to 4); any previous aortic repair, including any previous endovascular (thoracic endovascular aortic repair or endovascular abdominal aortic aneurysm repair) or open surgical procedure (thoracic aortic replacement, open repair for abdominal aortic aneurysm); staging (including patients who underwent > 1 procedures to complete aneurysm exclusion); setting of repairs (elective vs. urgent; including patients with rupture or symptomatic non-ruptured aortic aneurysm); presence of aortic rupture; and extent of the disease (type I to III TAAAs and proximal landing above zone 5 and distal landing below zone 9). Especially for the extent of the disease, the patients were matched according to aneurysm classification: juxtarenal, pararenal, or thoracoabdominal aortic aneurysm according to the Crawford criteria. In addition, the extent of coverage was assessed by matching patients according their proximal and distal sealing zones, including coverage above zone 5 or below and patients who needed distal sealing into the abdominal aorta (zone 9) or below (zone 10–11). Matching also included preservation of the collateral networks, including the presence of patent LSA and at least one hypogastric artery, preconditioning with intercostal embolization, and use or not of prophylactic CSFD. A logistic regression model was used for propensity score matching, applying 1:1 matching and a caliper width of 0.1. The *p*-value was considered significant when <0.05. No adjustment for missing data was performed. Statistical analysis was performed using SPSS 29.0 (IBM Corp., Armonk, NY, USA) for MacOS (Sonoma 14.5) software.

## 3. Results

### 3.1. Patient Cohort 

During the study period, 773 patients were managed with f/bEVAR. Among them, 65 presented SCI of any grade, leading to a rate of 8.4% for the total cohort, and 16 presented with Grade 3 (2.1%). Complete data on the intra-operative and early post-operative medical parameters were available in 51 cases. The pre-operative characteristics of the cohort are presented in Table 1. The mean age of the cohort was 69.8 ± 6.2 years and 39.2% were male. Thirty patients (58.8%) had a history of previous aortic repair, and 21 patients (41.2%) had a history of thoracic aortic repair. ASA score ≥ 3 was recorded in 64.7% of the cohort. 

The mean aortic diameter of the cohort was 67.4 ± 11 mm. Eleven patients (21.6%) were managed for chronic dissections, while 19 (37.3%) patients underwent urgent repair, 11 (21.6%) of them for aortic rupture and 8 (5.7%) for symptomatic aortic disease. TAAAs were managed in 34 (66.7%) cases; among them, 26 (51.0%) were types I to II. Staged repair was performed in 23 (45.1%) cases. FEVAR was performed in 12 (23.5%) patients and bEVAR in 32 (62.7%) patients. The remaining 7 patients were managed with devices incorporating both fenestrations and branches (13.7%). 

Regarding the extent of coverage, 29 (56.9%) patients needed coverage ≥ zone 4, while in terms of distal landing, 13 (25.5%) patients were managed with landing distally to zone 9 and 48 (94.1%) to zone 10 or below. The LSA was preserved in all patients (51;100%), while both hypogastric arteries were preserved in 48 patients (94.1%). Three patients presented with pre-existing bilateral hypogastric artery occlusion (5.9%). Six patients (11.8%) required hypogastric artery stenting for stenosis, as a preventive patency measure, while 3 (5.9%) were managed with iliac branched devices. No patient underwent intercostal or lumbar artery embolization as a measure of preconditioning. Regarding the peri-operative antithrombotic treatment, 29 (56.8%) were under single antiplatelet therapy with aspirin, 5 (9.8%) were under single antiplatelet therapy with clopidogrel, 8 (15.7%) were under double antiplatelet therapy, and 9 (17.6%) were under anticoagulant therapy.

### 3.2. Intra-Operative Characteristics among SCI and Grade 3 Patients

Technical success was achieved in all cases. Twenty-six (51.0%) patients had a prophylactic CSFD and 17 (33.3%) received therapeutic CSFD. The intra-operative parameters are presented in Table 2. The mean value of minimum systolic pressure among Grade 3 SCI patients was 88 mmHg, which was significantly lower than in patients with Grades 1 and 2 SCI (*p* = 0.02). A comparative analysis among patients with Grade 3 SCI vs. Grades 1 and 2 SCI is presented in Table 2.

Regarding the post-operative parameters, the mean glucose values at 24 and 48 h were 136 ± 19 mg/dL and 132 ± 18 mg/dL, respectively, and the mean hemoglobin values were 10.3 ± 0.9 mg/dL and 9.4 ± 0.8 mg/dL at 24 and 48 h, respectively. Twenty-three patients (45.1%) needed a transfusion post-operatively. The mean creatinine value at 24 h was 1.5 ± 0.5 mg/dL and the mean creatine value at 48 h was 1.6 ± 0.7 mg/dL (Table 3). The post-operative parameters of patients with late SCI are also presented in Table 3. Compared to early SCI patients, a significant difference was detected in terms of the need for transfusion (early: 56.8% vs. late: 14.3%, *p* = 0.006) and the mean creatinine value at 48 h (early 1.7 ± 0.8 mg/dL vs. late: 1.3 ± 0.9 mg/dL, *p* = 0.03).

### 3.3. ROC Analysis for Intra-Operative Parameters and Grade 3 SCI 

**Figure 1:**
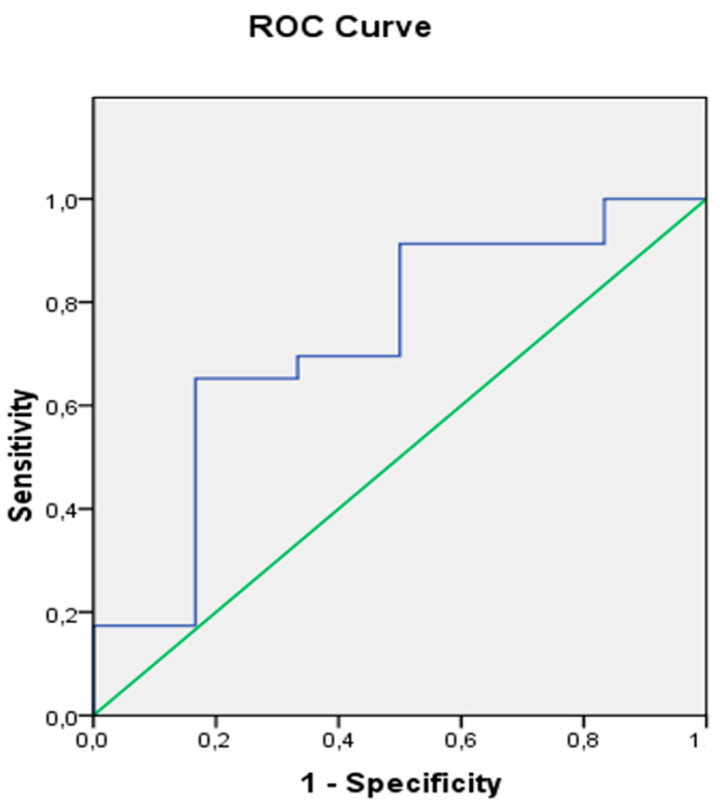
An intra-operative glucose value below 110 mg/dL was able to predict by 73% the prevention of Grade 3 spinal cord ischemia, with a sensitivity rate of 91% and specificity of 83%.

Of all intra-operative factors, only the mean intra-operative glucose and hemoglobin values provided an area under the curve (AUC) over 0.5. Specifically, a mean intra-operative glucose value < 110 mg/dL was a negative predictor for Grade 3 SCI (AUC: 0.73), with a sensitivity of 91% and specificity of 83% (Figure 1). 

**Figure 2:**
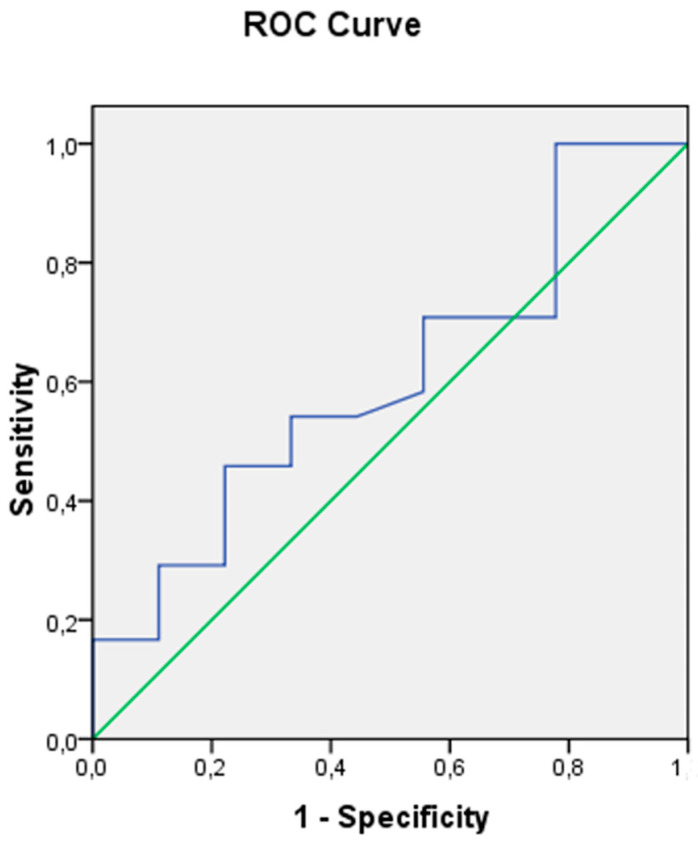
An intra-operative hemoglobin value over 8.5 mg/dL was able to predict by 61% the prevention of Grade 3 spinal cord ischemia, with a sensitivity rate of 83% and specificity of 78%.

A mean intra-operative hemoglobin value > 8.5 mg/dL was a negative predictor for Grade 3 SCI (AUC: 0.61), with a sensitivity of 83% and specificity of 78% (Figure 2).

### 3.4. Propensity Analysis on the Impact of Intra- and Early Post-Operative Factors on SCI 

Twenty-three patients of the SCI group were matched to 23 patients with similar pre-operative characteristics. Regarding the intra-operative parameters, no statistically significant difference was detected in terms of hemoglobin (max, min, and mean values; *p* = 0.76, *p* = 0.88, and *p* = 0.61, respectively) or need for transfusion (*p* = 0.09, Table 4). However, patients with SCI presented significantly higher glucose levels intra-operatively compared to non-SCI patients (glucose mean value: SCI: 150 ± 46 mg/dL vs. non-SCI: 122 ± 30 mg/dL, *p* = 0.005), as presented in Table 4. In addition to glucose, the ACT value (ACT mean value: SCI: 259 ± 31 s vs. non-SCI: 288 ± 28 s, *p* = 0.001) and volume input (SCI: 4030 ± 1430 mL vs. non-SCI: 3020 ± 1130 mL, *p* = 0.009) affected the evolution of SCI post-operatively (Table 4). 

The post-operative values of hemoglobin and creatinine were not significantly different between groups, as presented in Table 5, but more patients in the SCI group needed a transfusion (SCI: 52.5% vs. 4.3%, *p* < 0.001). Higher glucose levels were detected among patients with SCI, at 24 (SCI: 142 ± 30 mg/dL vs. non-SCI: 118 ± 26 mg/dL, p.004) and 48 h (SCI: 140 ± 29 mg/dL vs. non-SCI: 112 ± 20 mg/dL, *p* < 0.001, Table 5). No potential relationship between the post-operative values of hemoglobin, glucose, or creatine was detected between patients with late-onset SCI and non-SCI (Table 5). 

Regarding the post-operative outcomes, patients with SCI presented higher rates of AKI (SCI: 43.5% vs. non-SCI: 13.0%, *p* = 0.02) and bleeding events (SCI: 26.1% vs. non-SCI: 4.3%, *p* = 0.04). However, the need for reintervention due to bleeding was similar between groups (*p* = 0.07, Supplementary Table S1). The length of stay in the ICU (SCI: 11 days, IQR 7, range 2.30 vs. non-SCI: 3 days, IQR 2, range 1–13, *p* < 0.001) and the total length of hospital stay (SCI: 17 days, IQR: 20, range 2–65 vs. non-SCI: 13, IQR: 8, range 5–43, *p* = 0.02) were both significantly higher in the SCI group. 

## 4. Discussion

Despite the evolution of endovascular management of complex aortic aneurysms and increasing experience in the field, SCI is still a significant adverse event, affecting both the patient and health system [1,2]. Parameters such as the extent of disease, coverage, and status of the collateral network have been related to SCI evolution [20,21]. In addition to anatomic and technical factors, intra-operative persistent hypotension, hemoglobin, and glucose levels also seem to affect SCI rates [15,22]. Specifically, euglycemia, intra- and early post-operatively, has been directly related to SCI prevention [15]. This analysis showed that a glucose cut-off point at 110 mg/dL could be protective of Grade 3 SCI, while patients with SCI tended to have higher glucose levels intra- and early post-operatively. ACT value, volume input, and need for transfusion were also related to SCI. 

Current guidelines do not provide any recommendation on the management of these intra-operative parameters during complex endovascular procedures, despite previously reported findings in the literature and the fact that they could be considered as modifiable factors for SCI prevention [23,24]. However, it should be noted that specific recommendations on the intra-operative management of complex endovascular procedures do not exist and different aortic centers base their decisions on previous experience, local protocols, and discussions of multidisciplinary boards. Hemodynamic stability and hemoglobin levels over 8 mg/dL represent a rather common strategy among aortic centers [25].

Anemia has been detected as a negative predictor for post-operative morbidity and mortality after TEVAR, increasing the risk for 30-day death by 141%, while severe anemia has been related to lower survival rates within the first year of follow-up [26]. In addition, the need for transfusion has also been detected as a negative predictor not only for mortality but also for SCI evolution and cardiac adverse events after coverage of the descending thoracic aorta [27]. The current analysis is in accordance with the previously published literature, suggesting that need for transfusion may be predictive of SCI, while a hemoglobin level over 8.5 mg/dL is able to protect SCI patients against developing Grade 3 SCI. 

In addition, volume replacement seemed also to be a factor associated with worse outcomes regarding SCI. Despite the fact that intra-operative blood pressure was not detected as a relevant parameter, hemoglobin levels, the need for transfusion, and volume replacement may be able to construct a profile of a patient at risk for SCI, showing that the target of hemodynamic stability, including stable hemoglobin levels over 8.5 mg/dL, should represent a common baseline goal in all patients managed for complex aortic diseases [22,28]. The application of appropriate measures, such as quick recovery from hemodynamic instability and decrease of cerebrospinal fluid pressure, seem to lead to complete SCI resolution in bEVAR patients [28].

Euglycemia has been previously described as a protective factor for SCI [23]. High blood and cerebrospinal fluid glucose levels have both been associated with SCI, especially during the first post-operative day after bEVAR [23,29]. The current analysis confirms these findings not only by suggesting a threshold of 110 mg/dL as a targeted value to prevent Grade 3 SCI but also by detecting higher intra- and post-operative glucose levels among patients with SCI compared to matched non-SCI cases. Lowest value thresholds for SCI prevention could not be provided by the current cohort analysis, but further studies are needed. It should be noted that the presence of diabetes mellitus does not seem to play a role in SCI evolution after complex endovascular aortic procedures, as the underlying mechanism is rather guided by an acute self-limited phenomenon of insulin resistance, with a five-fold increase during the very early post-operative period [30]. Insulin infusion protocols have been proposed for patients needing extensive aortic coverage and seem to have a positive impact on SCI prevention compared to patients without tight glucose control during the early post-operative period [29].

Previous experience from TEVAR cases showed that appropriate heparinization can be protective for thromboembolic complications, even in urgent management of blunt thoracic aortic injuries [31]. In the current analysis, patients with SCI had a minimum ACT value at 219 sec, showing that fluctuations of ACT below the targeted values may be indicative of thrombotic events and strict control intra-operatively is mandatory for SCI prevention. However, heparin resistance in SCI patients cannot be excluded as a parallel mechanism leading to ischemic events, despite that it is not investigated in patients managed with f/bEVAR. Previous data showed that heparin resistance during cardiopulmonary bypass is rather a common phenomenon and also affects patients with normal antithrombin profile [32,33]. ACT values differed significantly between the SCI and non-SCI cohort, a finding indicating the potential involvement of thrombotic mechanisms in SCI development and highlighting the need for further investigation of this field. Previous studies showed that factors such as thrombospindin-1, released mainly by platelet a-granules, may play a role in hemorrhagic and ischemic complications of the central nervous system, including spinal cord injury, and may be able to predict their clinical evolution [34,35,36]. However, data focusing on the role of thrombospindin-1 in patients managed with endovascular aortic repair are lacking. In any case, ACT levels over 250 sec represent good practice, and strict control with repeated evaluation and additional heparin administration seem mandatory to prevent thromboembolic events during f/bEVAR.

The findings of this analysis highlight the multifactorial nature of SCI. Despite that a few anatomic and surgical parameters are already known to be related to SCI evolution, the use of prophylactic CSFD and medical parameters and treatment are still under investigation. This study aimed to highlight exactly the fact that a holistic and multidisciplinary approach is mandatory for complication prevention in cases undergoing complex endovascular management. Due to the small number of cases and the single-center data presented in this analysis, the generalizability of the findings should be viewed with caution. Future multicenter prospective or retrospective studies, including larger cohorts, could shed more light in the peri-operative management of f/bEVAR patients and the role of medical factors, such as glucose and hemoglobin values, heparin administration, and ACT targets, in SCI. Especially for glucose management, prospective randomized studies focusing on the use of insulin administration for strict glucose control intra- and post-operatively in patients at risk for SCI represent a topic of great interest [29]. 

### Limitations

The main limitations of the study are the small sample size and retrospective design. Especially, the retrospective nature of the study introduced significant confounders, selection bias, and hampered the ability to establish causality between factors and outcomes. The cohort represents the 10-year experience of a large volume aortic center; however, as this analysis is conducted using single-center data, generalizability in the real world should be made with caution. Despite the large number of cases managed in our center, data extraction from anesthesiologic reports was not feasible for all of them. Thus, reporting bias cannot be excluded. In addition, the small sample size after matching, especially for Grade 3 and late SCI patients, significantly affected the power of our analysis and, further, the robustness of our outcomes. The addition of various factors known to be related to SCI development after f/bEVAR affected the number of the included cases during matching but reassured the appropriate comparison between similar groups. Despite the fact that a matched cohort of non-SCI cases was used, parameters such as the presence of an endoleak, surgeon and anesthesiologist experience, and duration of the operation were not used as confounders. Medical treatment, including pre- and post-operative antithrombotic factors, genetic predisposition, and the potential impact of mechanisms of the pathways of inflammation and thrombosis cannot be excluded. Regarding the late SCI cohort, only 7 cases were matched to non-SCI cases, despite that the total number of late SCI events was 14. However, including all 14 late SCI patients and comparing them with 14 random non-SCI patients would introduce significant bias due to confounders, including the extent of the disease, the extent of coverage, status of the collateral network, etc. Only specific values (maximum, minimum, and mean) of the different potential factors for SCI evolution were collected, and the impact of intra-operative fluctuations was not investigated. In addition, the ROC analysis using the minimum glucose values available in this cohort provided an AUC = 0.47, hampering the predictability of the lowest glucose value in SCI evolution. Euglycemia (values within 70–110 mg/dL) should be targeted until further information in the literature is available. Investigation for heparin resistance does not represent a common clinical practice among patients managed with f/bEVAR and its impact on SCI could not be investigated or excluded. Further analyses on this topic could shed more light. The findings of this study should be examined under the potential presence of type-I statistical error due to the lack of correction for multiple hypotheses. 

## 5. Conclusions

SCI after f/bEVAR is of a multifactorial nature, with intra- and early post-operative parameters affecting outcomes. Intra- and early post-operative glucose levels are associated with SCI evolution and a targeted glucose level < 110 mg/dL may be protective for Grade 3 SCI.

## Figures and Tables

**Table 1:** The pre-operative characteristics of the cohort. Footnotes: ASA: American Society of Anesthesiologists; CABG: coronary-aorta bypass grafting; CAD: coronary artery disease; PCI: percutaneous coronary intervention.

Pre-Operative Characteristics (n; %, Mean ± Standard Deviation, Median, IQR)	SCI Cohort (51 Patients)
Age (years)	69.8 ± 6.2
Male	20 (39.2%)
CAD	10 (19.6%)
Myocardial infarction	3 (5.9%)
Heart failure	1 (1.9%)
CABG	1 (1.9%)
PCI	4 (7.8%)
Hypertension	27 (52.9%)
Dyslipidemia	6 (11.8%)
Tobacco use	17 (33.3%)
-Active tobacco use	10 (19.6%)
COPD	10 (19.6%)
Diabetes	7 (13.7%)
Chronic renal disease	14 (27.5%)
-Dialysis	1 (1.9%)
Stroke	5 (9.8%)
Peripheral arterial disease	4 (7.8%)
Any previous aortic repair	30 (58.8%)
-Abdominal aorta	14 (27.5%)
-Thoracic aorta	21 (41.2%)
ASA score	3 (IQR 0, range 1–4)
ASA score ≥ 3	33 (64.7%)
ASA score 4	5 (9.8%)

**Table 2:** Intra-operative parameters among the total cohort and Grade 3 SCI patients. Grade 3 SCI patients were compared to Grades 1 and 2 SCI cases. It should be noted that the mean value of minimum systolic pressure among Grade 3 SCI patients was below 90 mmHg, the threshold for hemodynamic instability identification, and significantly lower than in Grades 1 and 2 SCI patients. Footnotes: ACT: activating clotting time; Hgb: hemoglobin; RBC: red blood cells; UFH: unfractionated heparin; SCI: spinal cord ischemia.

Intra-Operative Parameters	SCI Cohort (51 Patients)	Grade 1 & 2 (37 Patients)	Grade 3 (14 Patients)	*p* Value
Hgb max (mg/dL)	11.2 ± 1.5	11.4 ± 1.9	10.9 ± 2.3	0.56
Hgb min (mg/dL)	9.4 ± 1.6	9.6 ± 2.0	9.4 ± 1.3	0.31
Hgb mean (mg/dL)	10.4 ± 1.6	10.6 ± 1.8	10.5 ± 1.5	0.31
Patients needing transfusion	24 (47.0%)	13 (35.1%)	11 (78.6%)	0.005
Transfused volume of RBC (mL)	490 ± 760	305 ± 442	974 ± 2156	0.32
Volume input (no blood products; mL)	4167 ± 1380	3793 ± 1146	5392 ± 3556	0.32
Glucose max (mg/dL)	187 ± 69	194 ± 126	165 ± 89	0.47
Glucose min (mg/dL)	129 ± 36	133 ± 44	113 ± 27	0.22
Glucose mean (mg/dL)	143 ± 39	149 ± 45	123 ± 42	0.21
Lactate max (mmol/L)	2.3 ± 1.7	2.4 ± 2.2	2.0 ± 1.8	0.32
Lactate min (mmol/L)	0.9 ± 0.4	1.0 ± 0.5	0.7 ± 0.2	0.06
Lactate mean (mmol/L)	1.4 ± 0.8	1.5 ± 1.0	1.3 ± 0.7	0.06
ACT max (s)	315 ± 32	309 ± 38	332 ± 57	0.03
ACT min (s)	223 ± 28	231 ± 28	200 ± 59	0.005
ACT mean (s)	268 ± 19	268 ± 25	268 ± 17	0.82
Administrated UFH (IU)	14,250 ± 5170	13,657 ± 5066	17,846 ± 9785	0.05
Systolic pressure max (mmHg)	149 ± 11	146 ± 10	158 ± 29	0.07
Systolic pressure min (mmHg)	96 ± 8	99 ± 6	88 ± 19	0.02
Systolic pressure mean (mmHg)	119 ± 6	119 ± 7	119 ± 9	0.98

**Table 3:** The early post-operative parameters among the total cohort and early vs. late SCI patients. Footnotes: Hgb: hemoglobin; SCI: spinal cord ischemia.

Early Post-Operative Parameters	SCI Cohort (51 Patients)	Early SCI (37 Patients)	Late SCI Group (14 Patients)	*p* Value
Hgb 24 h (mg/dL)	10.3 ± 0.9	10.2 ± 1.0	10.6 ± 1.5	0.37
Hgb 48 h (mg/dL)	9.4 ± 0.8	9.3 ± 0.9	9.7 ± 1.6	0.32
Patients needing transfusion	23 (45.1%)	21 (56.8%)	2 (14.3%)	0.006
Glucose 24 h (mg/dL)	136 ± 19	138 ± 23	130 ± 22	0.66
Glucose 48 h (mg/dL)	132 ± 18	133 ± 22	130 ± 28	0.88
Creatinine 24 h (mg/dL)	1.5 ± 0.5	1.5 ± 0.5	1.3 ± 0.9	0.07
Creatinine 48 h (mg/dL)	1.6 ± 0.7	1.7 ± 0.8	1.3 ± 0.9	0.03

**Table 4:** Comparative intra-operative parameters between matched SCI and non-SCI patients. Glucose and ACT seem to be predictors for SCI. Footnotes: ACT: activating clotting time; Hgb: hemoglobin; RBC: red blood cells; UFH: unfractionated heparin; SCI: spinal cord ischemia.

Intra-Operative Parameters	SCI Group (23 Patients)	Non-SCI Group (23 Patients)	*p*
Hgb max (mg/dL)	11.2 ± 1.7	11.7 ± 1.8	0.76
Hgb min (mg/dL)	9.5 ± 1.8	9.9 ± 1.9	0.88
Hgb mean (mg/dL)	10.4 ± 1.5	10.7 ± 1.8	0.61
Transfused volume of RBC (mL)	528 ± 293	155 ± 126	0.09
Volume input (mL)	4030 ± 1430	3020 ± 1130	0.009
Glucose max (mg/dL)	186 ± 107	138 ± 47	0.01
Glucose min (mg/dL)	130 ± 38	112 ± 23	0.04
Glucose mean (mg/dL)	150 ± 46	122 ± 30	0.005
Lactate max (mmol/L)	2.4 ± 1.9	1.2 ± 0.8	0.43
Lactate min (mmol/L)	1.0 ± 0.5	0.7 ± 0.2	0.57
Lactate mean (mmol/L)	1.6 ± 1.0	0.9 ± 0.5	0.25
ACT max (s)	304 ± 51	320 ± 32	0.43
ACT min (s)	219 ± 42	248 ± 25	0.01
ACT mean (s)	259 ± 31	288 ± 28	0.001
Administrated UFH (IU)	12,400 ± 7030	11,300 ± 3880	0.50
Systolic pressure max (mmHg)	152 ± 20	146 ± 17	0.47
Systolic pressure min (mmHg)	95 ± 8	96 ± 12	0.56
Systolic pressure mean (mmHg)	119 ± 9	121 ± 11	0.50

**Table 5:** Comparative early post-operative parameters between matched SCI and non-SCI patients and matched late SCI and non-SCI patients. Glucose levels and need for transfusion were related to SCI. Footnotes: Hgb: hemoglobin; SCI: spinal cord ischemia.

**Early Post-Operative Parameters**	**SCI Group (23 Patients)**	**Non-SCI Group (23 Patients)**	***p***
Hgb 24 h (mg/dL)	10.4 ± 0.9	10.1 ± 1.2	0.86
Hgb 48 h (mg/dL)	9.5 ± 1.1	9.1 ± 1.4	0.66
Patients needing transfusion	12 (52.2%)	1 (4.3%)	<0.001
Glucose 24 h (mg/dL)	142 ± 30	118 ± 26	0.004
Glucose 48 h (mg/dL)	140 ± 29	112 ± 20	<0.001
Creatinine 24 h (mg/dL)	1.5 ± 0.8	1.1 ± 0.5	0.37
Creatinine 48 h (mg/dL)	1.7 ± 1.1	1.1 ± 0.5	0.75
**Early post-operative Parameters**	**Late SCI group (7 patients)**	**Non-SCI group (7 patients)**	***p***
Hgb 24 h (mg/dL)	10.4 ± 1.9	10.7 ± 1.8	0.63
Hgb 48 h (mg/dL)	9.3 ± 2.0	8.5 ± 3.0	0.52
Patients needing transfusion	2 (28.6%)	0 (0.0%)	0.13
Glucose 24 h (mg/dL)	134 ± 37	116 ± 39	0.18
Glucose 48 h (mg/dL)	136 ± 50	118 ± 53	0.18
Creatinine 24 h (mg/dL)	1.6 ± 1.2	1.5 ± 1.0	0.92
Creatinine 48 h (mg/dL)	1.2 ± 0.9	1.3 ± 1.0	0.89

## Data Availability

The data presented in this study are available upon reasonable request from the corresponding author.

## Supplementary Materials

The following supporting information can be downloaded at: https://www.mdpi.com/article/10.3390/jcm13133978/s1, Table S1: Post-operative 30-day outcomes among patients with and without SCI.
