# Supplementary material for: Intra- and Early Post-Operative Factors Affecting Spinal Cord Ischemia in Patients Undergoing Fenestrated and Branched Endovascular Aortic Repair
Source: J Clin Med. 2024 Jul 8;13(13):3978. doi: 10.3390/jcm13133978 (PMC11242175; doi:10.3390/jcm13133978)
Supplement: Supplementary file 1 [file jcm-13-03978-s001.zip › jcm-3077331-supplementary.pdf]

**Supplementary Table S1.** Post-operative 30-day outcomes among patients with and without SCI evolution. Footnotes: ICU: intensive care unit; SCI: spinal cord ischemia

| Post-operative outcomes (n, %) | SCI group (23 patients) | Non-SCI group (23 patients) | P |
|---|---|---|---|
| Mortality | 2 (8.7%) | 0 (0.0%) | 0.14 |
| SCI | 23 (100%) | 0 (0.0%) | - |
| -Grade 1 | 4 (17.4%) | - | - |
| -Grade 2 | 14 (60.9%) | - | - |
| -Grade 3 | 5 (21.7%) | - | - |
| -Late evolution | 7 (30.4%) | - | - |
| -Recovery | 19 (82.6%) | - | - |
| -Partial | 12 (52.2%) | - | - |
| -Total | 7 (30.4%) | - | - |
| Stroke | 0 (0.0%) | 0 (0.0%) | - |
| Mesenteric ischemia | 2 (8.7%) | 0 (0.0%) | 0.14 |
| Myocardial infarction | 0 (0.0%) | 0 (0.0%) | - |
| Acute kidney injury | 10 (43.5%) | 3 (13.0%) | 0.02 |
| Bleeding | 6 (26.1%) | 1 (4.3%) | 0.04 |
| -Needing reintervention | 3 (13.0%) | 0 (0.0%) | 0.07 |
| ICU length (days) | 11 (IQR 7, range 2-30) | 3 (IQR 2, range 1-13) | <0.001 |
| Length of hospital stay (days) | 17 (IQR 20, range 2-65) | 13 (IQR 8, range 5-43) | 0.02 |
